# Expression Profiling of Primary and Metastatic Ovarian Tumors Reveals Differences Indicative of Aggressive Disease

**DOI:** 10.1371/journal.pone.0094476

**Published:** 2014-04-14

**Authors:** Alexander S. Brodsky, Andrew Fischer, Daniel H. Miller, Souriya Vang, Shannon MacLaughlan, Hsin-Ta Wu, Jovian Yu, Margaret Steinhoff, Colin Collins, Peter J. S. Smith, Benjamin J. Raphael, Laurent Brard

**Affiliations:** 1 Department of Molecular Biology, Cell Biology, and Biochemistry, Brown University, Providence, Rhode Island, United States of America; 2 Center for Computational Molecular Biology, Brown University, Providence, Rhode Island, United States of America; 3 Center for Genomics and Proteomics, Brown University, Providence, Rhode Island, United States of America; 4 Department of Computer Science, Brown University, Providence, Rhode Island, United States of America; 5 Program in Women's Oncology, Department of Obstetrics and Gynecology, Women and Infants’ Hospital, Alpert Medical School of Brown University, Providence, Rhode Island, United States of America; 6 Department of Pathology, Women and Infants’ Hospital, Alpert Medical School of Brown University, Providence, Rhode Island, United States of America; 7 Vancouver Prostate Centre, Vancouver, Canada; 8 Institute for Life Sciences, University of Southampton, Southampton, England; 9 Department of Obstetrics and Gynecology, Division of Gynecologic Oncology, Southern Illinois University School of Medicine, Springfield, Illinois, United States of America; Kinghorn Cancer Centre, Garvan Institute of Medical Research, Australia

## Abstract

The behavior and genetics of serous epithelial ovarian cancer (EOC) metastasis, the form of the disease lethal to patients, is poorly understood. The unique properties of metastases are critical to understand to improve treatments of the disease that remains in patients after debulking surgery. We sought to identify the genetic and phenotypic landscape of metastatic progression of EOC to understand how metastases compare to primary tumors. DNA copy number and mRNA expression differences between matched primary human tumors and omental metastases, collected at the same time during debulking surgery before chemotherapy, were measured using microarrays. qPCR and immunohistochemistry validated findings. Pathway analysis of mRNA expression revealed metastatic cancer cells are more proliferative and less apoptotic than primary tumors, perhaps explaining the aggressive nature of these lesions. Most cases had copy number aberrations (CNAs) that differed between primary and metastatic tumors, but we did not detect CNAs that are recurrent across cases. A six gene expression signature distinguishes primary from metastatic tumors and predicts overall survival in independent datasets. The genetic differences between primary and metastatic tumors, yet common expression changes, suggest that the major clone in metastases is not the same as in primary tumors, but the cancer cells adapt to the omentum similarly. Together, these data highlight how ovarian tumors develop into a distinct, more aggressive metastatic state that should be considered for therapy development.

## Introduction

Serous Epithelial Ovarian Cancer (EOC) is an aggressive disease for which there are few effective biomarkers and therapies. EOC is often diagnosed after tumor cells have disseminated within the peritoneal cavity [1] and metastases account for the majority of disease-related deaths. Despite its vital role in disease progression, however, the features required for ovarian cancer metastasis remain poorly understood [1]. Ovarian tumors do not typically spread through a hematogenous route, but rather shed from the primary tumor and enter the peritoneal fluid. Primary ovarian tumors typically spread within the peritoneal cavity, most often to the omentum.

The purpose of this study was to identify features that may be important in establishing metastases. As cancer cells metastasize, specific cancer cells with distinct genomes and phenotypes may be selected. Comparing primary and metastatic tumors has generated important insights into disease progression in both animal models [2] and in patients [3]. To improve treatment of metastatic disease, it is vital to understand the genes and pathways expressed in metastases, as many genes have the potential to contribute to aggressive phenotypes.

mRNA expression data using early generation microarrays suggest there are few significant expression differences between omental lesions and primary tumors [4]–[6]. However, numerous studies testing specific functions observe differential expression of factors between primary tumors and metastases including MMPs [7], [8] and integrins [9]. Copy number changes, large structural variants, and point mutations identified by next-generation sequencing suggest that specific genetic differences are found in metastases compared to primary tumors from the same patient [10]–[13].

We observe significant differences between the primary and omental metastatic tumors by gene expression microarray analysis. We found that the copy number alterations that differ between matched primary and metastatic tumors do not explain the recurring expression differences that define common features of metastasis. Up-regulated signaling pathways, including TGFβ signaling, suggest that tumor cells are adapting to the new omental environment. qPCR and immunohistochemistry support the microarray findings that metastases appear to be more proliferative and have less apoptotic cells than primary tumors. We define a “metastatic expression signature” of the most significantly differentially expressed genes between primary and metastatic tumors. This signature identifies poor prognosis patients by Kaplan-Meier analysis in two large independent primary tumor datasets. Bootstrapping demonstrates that this six gene signature is among the top performing of all possible six gene combinations. In sum, these data suggest that metastatic tumors progress into a more aggressive state distinct from many primary tumors and may prove to be more indicative of the disease that needs to be treated.

## Materials and Methods

### Ethics Statement

Informed consent was obtained from all study participants prior to study entry by appropriately trained study personnel. After obtaining written consent, primary ovarian tumor and an omental metastatic tumor were collected from each de-identified cases using protocol #08-0095 approved by the Institutional Review Board of the Women’s and Infants Hospital of Rhode Island.

### Patient and Sample Collection

We identified 19 matched primary and omental metastatic tumor specimens from patients with serous adenocarcinomas including 14 serous epithelial ovarian, 4 serous epithelial fallopian tube and one serous epithelial primary peritoneal (Figure S1). All patients are post-menopausal and had metastatic disease (Table S1). All patients were over age 55, stage III or later, and all tumors were chemotherapy-naïve. A pathologist examined all specimens (MS). Samples were frozen in liquid nitrogen within ten minutes after extraction. Primary and metastatic tumors were collected during the same initial debulking surgery before chemotherapy. Only regions with >70% of cancer cells as determined by hematoxylin and eosin (H&E) staining were selected for analysis.

### RNA isolation and Affymetrix microarrays

Tumor tissue with >70% cancer cells was homogenized with a Tekmar Tissumizer (Cincinnati, OH). RNA was purified using miRNeasy kit (Qiagen, Valencia, CA). Nugen WT-Ovation Pico kit with the WT-Ovation Exon Module was used to prepare the RNA for Affymetrix Human Gene St v1.0 microarrays following manufacturer instructions in the Brown University Center for Genomics and Proteomics core facility. Data were quantile normalized and signals estimated using Robust Multi-array Average (RMA). Genes with consistent signal below the lowest quartile were removed. Data are deposited to the Gene Expression Omnibus in series GSE30587.

### DNA purification and copy number analysis

DNA was isolated using Qiagen DNeasy Blood and Tissue Kit following manufacturer’s instructions. DNA quality was determined on an agarose gel. DNA was prepared for Agilent 180K CGH microarrays using the Roche NimbleGen (Madison, WI) enzymatic labeling protocol using random nonamers and hybridized following manufacturer’s protocols at the Microarray Centre at the Prostate Centre in Vancouver, British Columbia, Canada. Each sample was hybridized with Promega (Madison, WI) female reference DNA. The log10 copy number ratios were smoothed using a standard deviation-based outlier detection method and segmented using Circular Binary Segmentation (CBS) [14] as implemented in the R package DNAcopy (‘smooth.region = 10’ for the smoothing method, ‘alpha = 0.05’ for the segmentation, and default parameters for all other arguments). A log10 copy number for each gene was computed by averaging the smoothed and segmented log10 ratios for each probe located within the gene region. Only genes that contained three or more probes were considered.

### Quantitative Real-Time PCR

Equal amounts of total RNA were reverse transcribed using Superscript III and random hexamers (Invitrogen, Carlsbad, CA). Resulting cDNA was renormalized using Quant-iT PicoGreen (Invitrogen) before mixing with Power SYBR Green PCR Master Mix (Applied Biosystems, Foster City, CA). Reactions were performed in an Applied Biosystems 7900HT Fast Real-Time PCR System. Primer sequences are listed in Table S2.

### Immunohistochemistry

4 µm slices of formalin fixed paraffin embedded (FFPE) tissue were prepared from each tumor. Proliferation index was determined by staining with anti-Ki-67 antibody (Dako, Cat # IS626). Ki-67 percentage was evaluated manually by comparison with a schematic representation of various percentages of positive-staining nuclei. Quantification of apoptosis in primary and metastatic serous epithelial carcinoma was performed with a Terminal deoxynucleotidyl transferase dUTP Nick End Labeling assay (TUNEL). Each slice was stained with ApopTag Plus Peroxidase In Situ Apoptosis Kit (Millipore, Billerica, MA). Slices were imaged with a Photometrics CoolSNAP camera (Photometrics, Tucson, AZ) under 10× magnification and assessed for positive apoptosis staining. An apoptotic index was calculated as the total number of apoptotic cells divided by the total number of tumor cells from 12 images across each slice. The distribution of percent apoptotic and Ki-67 cells is not normal, but is log-normal as determined by a Shapiro-Wilk test and quantile-quantile plots. Paired t-tests were calculated after log transformation in R.

### Bioinformatics and Survival Analysis

Hierarchical clustering was performed in Gene-E using Pearson correlation to calculate distances [15]. Survival analysis including the Cox Proportional Hazards model, Kaplan-Meier analysis, and statistical tests including Student’s t-test were performed in R. All TCGA data were downloaded from the TCGA data portal using the published dataset freeze. All TCGA data include primary ovarian tumors only. The Australian Oncology Group microarray data for ovarian tumors, GSE9891, was downloaded from GEO and processed using RMA.

## Results

### Identification of Recurring Differentially Expressed Genes

To determine if there are recurring phenotypic changes between primary and metastatic tumors, we measured the gene expression profiles in nine of the twelve pairs of primary and metastatic tumors used for copy number analysis with sufficient RNA quality for analysis (Table S1A). Three samples had poor RNA quality and were not considered further for RNA analysis.

77 up-regulated and 10 down-regulated genes between matched primary and omental tumors have average log2 fold changes >0.9 and q<0.25 in a paired t-test across the cohort (Table S3). The low number of differentially expressed genes across all cases by these criteria is similar to past studies [4], [6], [16] and may be because of interpatient variation or because of the relatively low sensitivity of microarrays, or may be because primary and metastatic tumors are very similar. However, qPCR of genes of interest suggests significant differential expression for many genes (Figure S2).

### Metastatic tumors are more proliferative and anti-apoptotic

**Figure 1:**
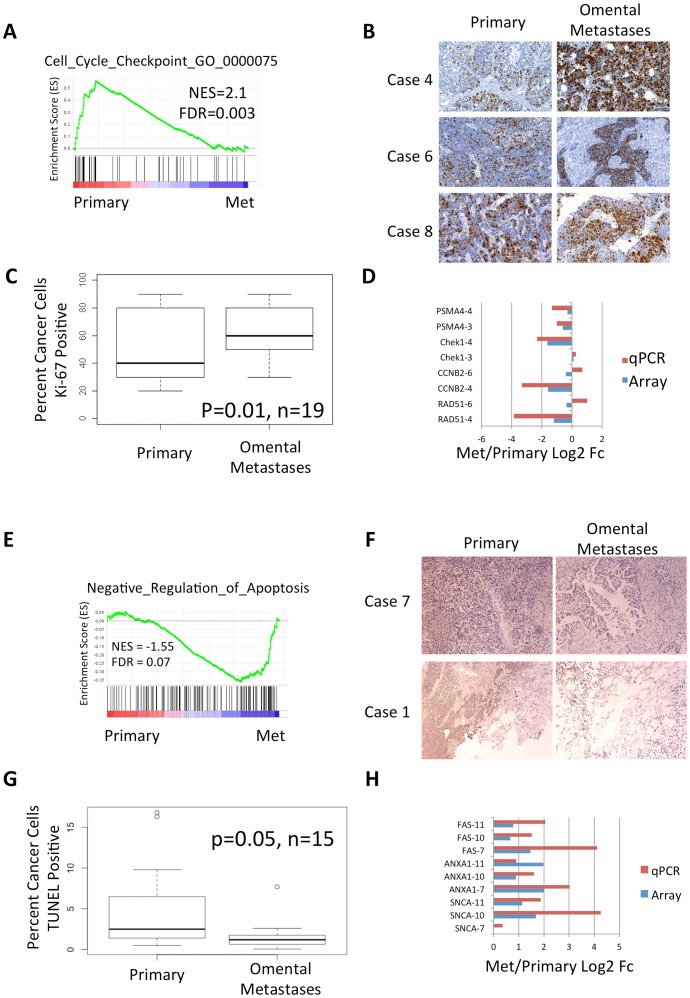
Metastases are more proliferative and include less apoptotic cells than matched primary tumors. **A.** GSEA enrichment plot suggests Reactome G2/M cell cycle checkpoints including Chek1, CCNB2, and BUB1 are expressed higher in primary tumors. **B.** Representative immunohistochemical staining of Ki-67 from three cases suggest higher Ki-67 staining in omental metastases. **C.** Box plot of the percent cells with positive Ki-67 staining. Paired t-test suggests significant differences between omental metastases and primary tumors from 19 cases. **D.** qPCR validation of cell cycle checkpoints. Note that genes with significant >1.8 fold changes in the array validate in those same tumors by qPCR. Genes with small expression changes measured by either the array or qPCR may be noisy in those tumors. **E.** GSEA enrichment plot suggests that negative regulators of apoptosis are up-regulated in metastases. **F.** TUNEL staining of two representative cases shows increased TUNEL signal in metastases. **G.** Box plot of the percent of cancer cells as determined by H&E staining with positive TUNEL staining. Paired t-test suggests significance.

In order to investigate differential expression of pathways between primary and metastatic tumors, we employed Gene Set Enrichment Analysis (GSEA) which does not depend on arbitrary thresholds [17]. We observed higher expression in metastases of cell communication, cell adhesion, and extracellular matrix receptor interactions, consistent with expected changes necessary for colonizing and establishing metastases (Table S4). In primary tumors, we observe higher expression of cell cycle and DNA repair factors, suggesting changes in proliferation rates (Table S4, Figure S3). In particular, GSEA suggests that G2/M checkpoint factors are more highly expressed in primary tumors compared to metastases (Figure 1A). We validated the expression levels of a subset of these genes by qRT-PCR (Figure 1D). We tested this hypothesis by staining for Ki-67 in each pair of matched tumors from 19 cases (Figure 1B, 1C and Table S5). These 19 cases include the 9 tested by microarray and an additional 10 cases (Table S1C). Ki-67 showed higher staining in metastases in 12/19 cases, including 6/9 of the cases used for expression microarrays, suggesting that omental metastases are often proliferating more rapidly than their matched primary tumors (p = 0.01, paired t-test), at the time of their collection.

To determine which expression changes may originate from cancer cells, we identified the genes with expression changes that correlate with the change in Ki-67 immunostaining. We examined the top 250 genes, all with Pearson correlations >0.72, in DAVID [18], and observed significant enrichment of cell cycle, M phase, cell cycle progress, and DNA replication genes (Table S6). This correlation analysis suggests that many of the cell cycle expression changes observed by microarray originate from cancer cells and not tumor infiltrating cells.

Higher expression of pro-survival genes is observed in the omental lesions including MYC, IRF1, BCL2L2, and TNFSF10 leading to biased expression of the gene ontology anti-apoptosis gene set as determined by GSEA (Figure 1B). Consistent with these gene expression changes, decreased TUNEL staining is observed in 12/15 tested metastases compared to primary tumors, including 7/9 of the cases measured on the microarrays (p = 0.05, t-test). Together with the Ki-67 observations (Figure 1A) these data suggest that cancer cells in metastases are often more proliferative and may be more inherently resistant to cell death induced by chemotherapeutics. We conclude that ovarian metastases adapt to the new omental environment in a more anti-apoptotic and proliferative state compared to primary tumors.

Expression levels of a number of growth pathways in metastases compared to primary tumors differ between primary and metastatic tumors, consistent with increased proliferation and reduced apoptosis (Table S4). Higher expression of Axin2, DKK2, NKD1, and NKD2 contribute to enrichment of WNT/β-Catenin signaling in metastases (Figure S3) consistent with recent studies [19], [20]. Other pathways linked to aggressive cancer include increased calcium signaling (NES = 2.03, FDR = 0.003), and increased Jak-Stat, Stat3, and NOTCH signaling in metastases (Table S4).

### Metastatic signature identifies more aggressive tumors

**Figure 2:**
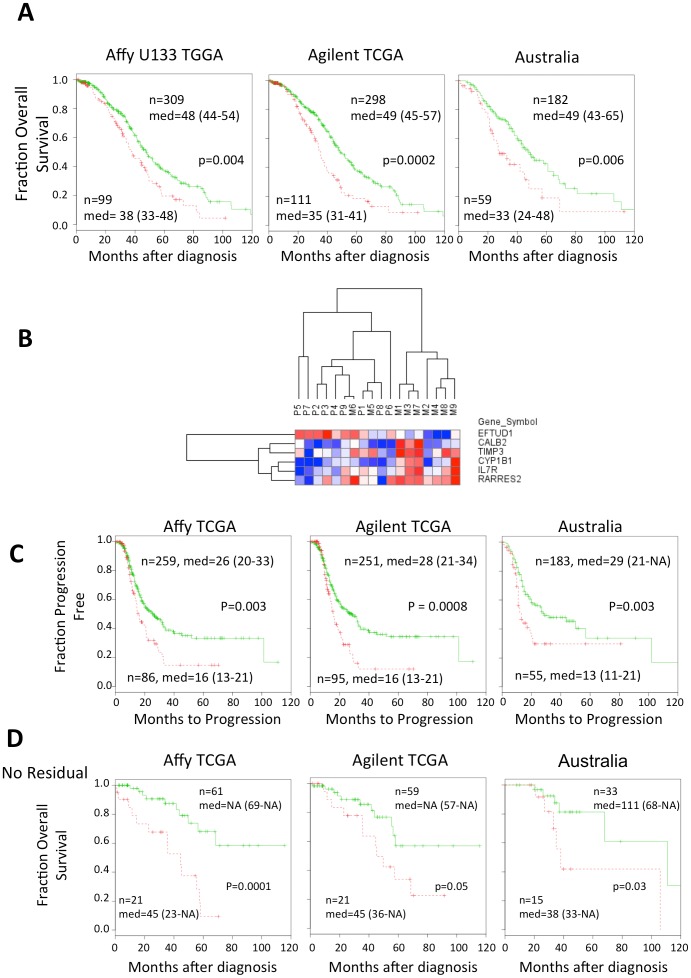
Six gene metastasis signature distinguishes aggressive disease in patients and primary and metastatic tumors. **A.** Red dotted lines indicate high risk group and the green line indicates the low risk group. For each patient’s tumor, a point was given for each metastasis gene for which higher than median expression was associated with longer survival, and vice versa. Tumors with more than three points were classified as high risk. Only patients who were treated with platinum or taxane chemotherapy were included. Two microarray platforms from TCGA were evaluated (Affymetrix U133A and the Agilent Custom 244K). A second independent dataset on Affymetrix U133 Plus 2 microarrays from the Australian Oncology group was also evaluated [18], [24]. The median survival is shown with 95% confidence levels in parentheses. **B.** Hierarchical clustering of the six gene metastasis signature separates the primary and metastatic tumors. Red indicates higher and blue lower expression. **C.** The six gene metastatic signature distinguishes more aggressive disease using time to progression as a metric. **D.** The six gene signature predicts survival for cases with no residual disease. Cases in each dataset with no macro disease after surgery were gathered and evaluated for overall survival.

Expression in primary tumors has long been associated with metastatic potential [1], [21]. We hypothesized that transcripts with significant expression changes to metastases are indicators of aggressive disease as metastases represent a more advanced stage of disease. To test this hypothesis, we identified transcripts that are differentially expressed between primary and metastatic tumors and also predict overall survival in two independent datasets in primary tumors. We started with the 87 genes that are the most differentially regulated (p<0.05, FDR<0.25, average Fc>0.9, Table S3). Using a scoring system similar to Kang *et al.* [11], [13], [22], genes were initially evaluated using the Cox proportional hazards model to determine if they are associated with survival in the TCGA Affymetrix U133A microarray data. TCGA data evaluated only primary tumors. For genes expressed higher in metastases compared to primary tumors, a point was assigned when the expression was higher than the median. For genes expressed lower in metastases compared to primary tumors, a point was assigned when the expression was lower than the median. After summing the points, cases with higher scores, >3 points, constituted the high score group and cases with ≤ 3 points were classified as the low score group in Kaplan-Meier analysis. Six of the twelve genes significantly distinguish high and low risk patients in three datasets: TCGA Affymetrix U133A microarrays, TCGA Agilent Custom 244K microarrays and Affymetrix U133 Plus 2 microarrays from the Australian Oncology Group [23], [24] (Figure 2A). We find that the two TCGA microarray platforms and decided to keep them separate to identify transcripts associated with survival in each dataset independently. These same six genes separate primary and metastatic tumors by hierarchical clustering with good specificity (Figure 2B). As another test of the six gene metastatic expression signature, we tested its ability to classify patients based on progression-free survival (PFS). Figure 2C shows the six gene signature can distinguish high and low risk patients based on PFS.

To further test the ability of the six gene classifier to distinguish high and low risk patients, we evaluated the relationship of the metastatic gene signature with residual disease. Residual disease is associated with overall survival [4]–[6], [25], [26], with low survival for patients with higher levels of macroscopic residual disease after debulking surgery. However, patients considered to have no residual disease after debulking surgery have an intermediate level of survival. Patients with no observable residual disease, may then presumably be subject to the innate aggressiveness of the disease and response to chemotherapy, and not other variables such as the location of the tumor and the ability to remove tumors surgically. The six gene signature separates the high and low risk patients with no macro-disease across all datasets very robustly (Fig. 2D). The six gene signature does not separate high and low risk patients with some residual disease as the effects of residual disease apparently supersede the signature’s ability to detect aggressive disease in the survival data. These observations suggest that the six gene signature identifies primary tumors that are the most aggressive.

Recent studies have suggested that there are many gene lists that are strong predictors, even across multiple datasets [7], [8], [27]. To determine if the six gene classifier is unique, or if there are multiple combinations of six expressed genes that can distinguish high and low risk patients, we evaluated >10,000 random sets of six genes in the Affymetrix TCGA data. We find that using the same parameters, that very few random gene lists of equivalent size are predictors (Table 1). Thus, the metastatic gene list is among the most significant possible combinations of six genes able to classify patients in these datasets.

**Table 1:** Specificity of metastatic six gene signature.

Dataset	P-value of 6 gene signature	P-value relative to random sets
Affymetrix TCGA	0.004	0.001 (n = 10,729)
Agilent TCGA	0.0002	0.0008 (n = 10,054)
Australia	0.006	0.0025 (n = 10,000)

P-values are calculated by determining the number of random gene sets with lower p-values than the metastatic gene signature using the log-rank test of Kaplan Meier analysis divided by the number of random sets tested as indicated in parentheses.

The six genes in the metastatic expression signature are shown in Table 2. IL7R is highly expressed in T-cells and thus may be associated with infiltrating T-cells, whose presence is higher in many of the metastatic tumors in our study as indicated by higher expression of other T-cell markers such as CD3 (data not shown). Infiltrating immune cells in primary ovarian tumors are associated with a metastatic phenotype and poor survival [9], [28]–[31]. The other five genes in the metastatic expression signature have not been directly linked with ovarian cancer to date.

**Table 2:** List of genes in six gene metastatic signature.

Gene	Refseq ID	Gene Name	Function
CALB2	NM_001740	Calbindin 2	Cell junction, calcium binding
CYP1B1	NM_000104	Cytochrome P450, family 1, subfamily B, polypeptide 1	Cholesterol and steroid metabolism
EFTUD1	NM_024580	Elongation factor Tu, GTP binding domain containing 1	Translation elongation factor
IL7R	NM_002185	Interleukin 7 receptor	Plays a critical role in the V(D)J recombination during lymphocyte development.
RARRES2	NM_000362	Retinoic acid receptor responder 2	Initiates chemotaxis via the ChemR23 G protein-coupled seven-transmembrane domain ligand.
TIMP3	NM_000362	TIMP metallopeptidase inhibitor 3	matrix metalloproteinase inhibitor

### Genetically diverse cancer cells establish omental lesions with similar phenotypes

**Figure 3:**
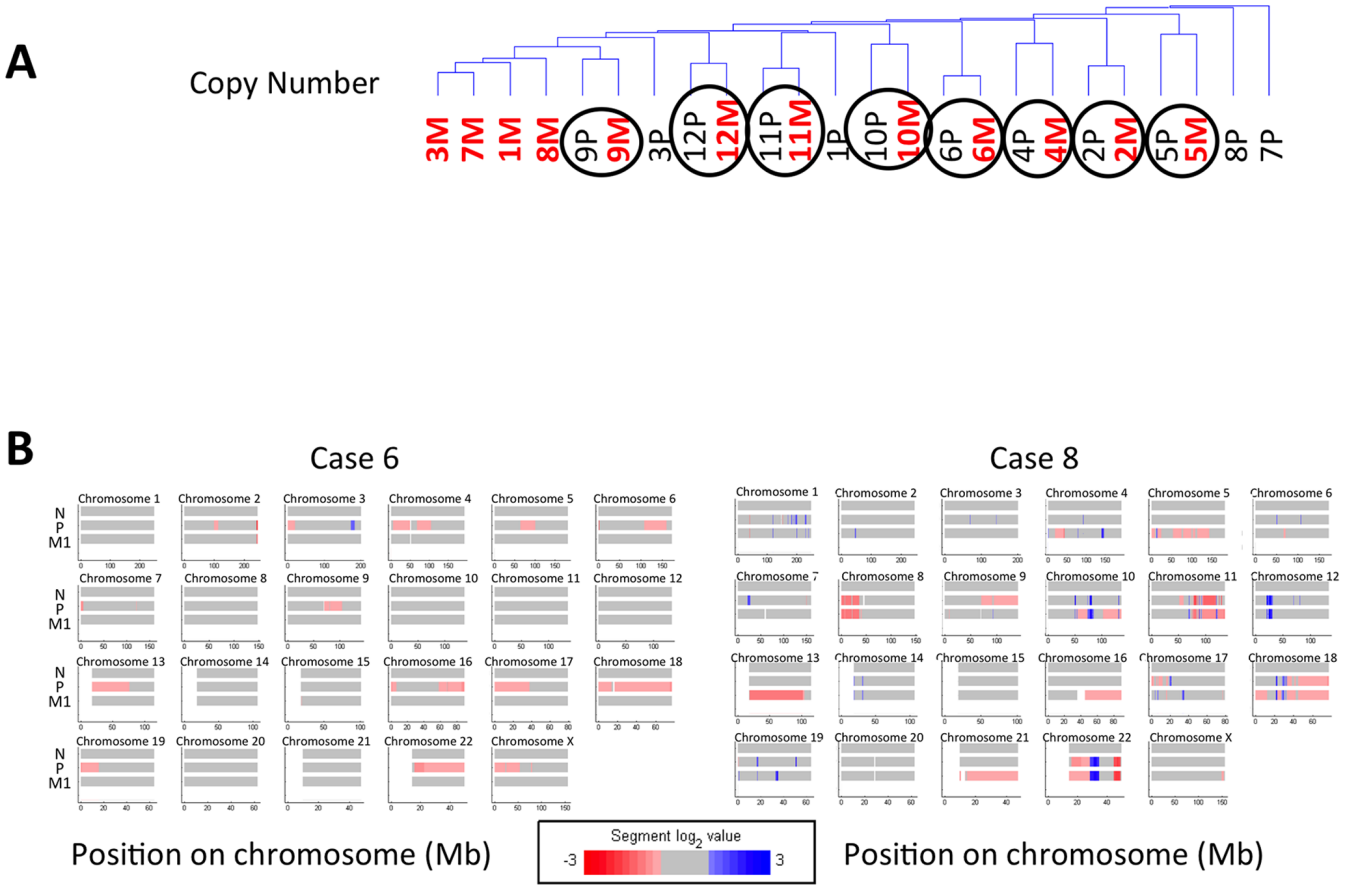
Hierarchical clustering of copy number aberrations and mRNA expression. **A.** Most patients cluster by their copy number aberrations (CNAs). The circles indicate matched pairs of primary and metastatic tumors that cluster together with similar patterns of CNAs. **B.** Representative heat maps of the copy number data from two cases. Many CNAs are found in both primary and metastatic tumors.

To determine if copy number aberrations contribute to the recurring gene expression changes, we evaluated DNA copy number from 12 matched primary and omental metastases using Agilent 180K CGH microarrays (Figure 3A). We performed hierarchical clustering to investigate the relationship among 24 tumors from 12 patients based on their genomic alterations. These data suggest that 8/12 primary-met pairs are more genetically similar to each other than the other tumors, consistent with a clonal expansion model [2], [23] and patient specific evolutionary trajectories [32]. Notably, almost every region of the genome is represented in the cumulative spectrum of CNAs observed in the collection of tumors, consistent with the known extensive genomic instability of EOC [3], [33] (Figure S4). Many CNAs are similar between the primary and metastatic tumor, as suggested by hierarchical clustering, however, notable differences are observed in each patient suggesting the metastases are genetically distinct from the primary tumor (Figure 3B).

Metastatic specific CNAs may indicate DNA copy number changes that are necessary to establish metastases. To determine if any metastatic specific CNAs are recurring, which may indicate functional importance, segmented regions were mapped to genes and a log2 ratio score for each gene was calculated. Genes in amplified or copy loss regions with high or low expression were selected. We observe one amplified gene, ERBB2, unique to metastases and four genes uniquely amplified in primary tumors, and not in omental lesions, in at least two patients where the expression and copy number are correlated (Pearson>0.7) (PDCD10, ZNF507, DTNA, AMN1). Each of these genes has lower expression in all metastases, consistent with the role of copy number in modulating expression. However, a recent study examining ERBB2 expression and amplification, did not detect noticeable differences between primary and omental metastases, perhaps because of significant intratumoral heterogeneity [14], [34]. These data suggest that few, if any, specific CNAs, unique to metastases, are providing a selective advantage to establish metastases in the major clone in the omental lesions. Alternatively, the primary and metastatic tumors may be so heterogeneous that they are not dominated by a single clone detectable in bulk tumor samples. Together, these observations suggest that gene expression may be more indicative of the key features for development of aggressive of ovarian cancer, than distinct genetic changes, or selection of specific clones.

## Discussion

Our key findings show that metastatic and primary tumors have different expression profiles reflecting their proliferation and apoptosis status. On the other hand, copy number analysis did not reveal recurrent genetic changes in this cohort, suggesting that the dominant clone in metastases may differ from the primary tumor, or that these tumors have high intratumoral heterogeneity. The expression changes indicative of aggressive disease between primary tumors and omental lesions are common and important for disease progression. Importantly, genes differentially expressed between primary tumors and omental lesions identify a predictive expression signature of patient outcome, suggesting the functional importance of these genes in metastasis and disease progression. Together, these observations support the idea that ovarian primary tumors encode significant metastatic potential.

Some studies suggest ovarian primary and metastatic tumors are very similar by expression analysis [4], [6], [35], while others suggest they differ [15], [16]. We conclude that these tumors differ in key features as supported by GSEA pathway analysis, IHC of Ki-67, apoptosis analysis, and the identification of the six gene expression signature. Our approach differs from past studies because of the application of GSEA, which does not consider arbitrary thresholds to identify differentially expressed pathways [17], leading to increased sensitivity. Second, we validate the GSEA analysis predictions with Ki-67 immunohistochemistry and TUNEL staining in the tested cases and an extended set of cases. Third, we identify a six gene signature that separates primary and metastatic tumors and identifies aggressive disease in two independent primary tumor datasets. Together, these observations support the conclusion that omental metastases have significantly different expression profiles than primary ovarian tumors, and these expression differences identify key features of aggressive disease. Importantly, these data may help explain why omental metastases are aggressive as they are more proliferative and are more resistant to cell death than primary tumors, similar to cancer cells in 3D culture environments or cells that have survived detached in culture for a prolonged period. These observations are consistent with the concept that disseminating cancer cells can become more anti-apoptotic during metastasis [9], [36], [37]. Future examination of larger patient cohorts to test if omental metastatic specific changes indicate patient survival better than primary tumors can help address these questions.

For many of the identified networks, we cannot strongly conclude that the expression changes are due to adaptation of the cancer cells or changes in the type and number of infiltrating cells. Our evaluation by immunohistochemistry and correlation of expression with Ki-67 signal does suggest that many of the expression changes originate from cancer cells. Nonetheless, some signal from infiltrating cells is present as evidenced by increased expression of CD3 and IL7R in metastases that likely originate from T cells. Because most large tumor characterization studies also use the same criteria as applied here to collect >70% bulk tumor, we were able to derive a predictive expression signature that includes genes that likely originate from both cancer and infiltrating cells.

The scoring system used here is flexible, as contributions from different sets of genes can be used to identify the high-risk patients for a general metastatic signature, allowing for some variation in the tumors in individual patients. We propose that the full 87 gene signature can be used as an initial set of genes to identify and to refine a metastatic gene signature that can distinguish primary and metastatic tumors as well as high risk patients in a wide-range of survival data. We find that random selections of gene sets rarely are associated with survival unlike the situation in breast cancer (Table 1). This could be because proliferation and the cell cycle based signatures do not predict survival in ovarian cancer as well as they do in breast cancer [6], [27], [38].

These data suggest that a variety of cancer genomes can lead to the same metastatic phenotype, defined by common expression patterns. We observe many common expression changes between primary and metastatic tumors, but no common enrichment of specific copy number changes. The diversity of genomes between primary and metastatic tumors in this cohort may be due to intratumoral or interpatient genetic heterogeneity or both. The diversity observed in this study is consistent with sequencing analysis of primary and metastatic ovarian tumors that found significant patient heterogeneity [32]. Ovarian cancer is a very genetically heterogeneous disease, but the phenotypes needed to metastasize appear to more common suggesting that targeting phenotypes may treat large patient populations than most genetic based approaches.

These observations highlight the changing state of ovarian tumors that should be considered as various treatment options are developed. Our data support the hypothesis that metastases are distinguishable from primary tumors, likely influenced by the omental microenvironment. This hypothesis suggests that deeper examination of metastases is necessary to treat the disease, as distinct pathways may be activated in metastases.

Understanding the molecular features unique to omental lesions may be critical to understand and to improve treatment of advanced ovarian cancer. These data should inspire future studies to address the function of these metastatic networks and how they may be important in sustaining the tumors and driving further metastasis. Future studies examining which signaling pathways or transcription factors cause the observed expression changes will be important to understand the regulation of these behaviors. Future maturation of the clinical data and larger patient cohorts will also likely provide more insights into the connections between metastasis, drug response, and patient outcomes.

## Supporting Information

Figure S1 **Tumors are of ovarian origin and are serous epithelial as indicated from examination of H&E and cytokeratin staining.** Representative H&E staining of two representative cases. CA125 and cytokeratin staining of one case is consistent with ovarian tumor origins.
(PPTX)

Figure S2 **Microarray and qPCR measurements generally agree.** qPCR validation of genes primary and metastatic tumors from two cases. Not all these genes have q<0.1.
(PPTX)

Figure S3 **Enriched pathways reveal common features of metastases.** GSEA enrichment plot suggests that WNT/β-Catenin signaling from the Signaling Transduction database and double strand break repair gene ontology are up-regulated in metastases compared to primary tumors.
(PPTX)

Figure S4 **Summary of all CNAs in all 12 cases.** Most of the genome is subject to copy number aberrations consistent with extensive genomic instability of serous ovarian cancer. Green is amplification, red is copy loss.
(PPTX)

Table S1 A. Cases used for mRNA microarrays. B. Cases used for CGH microrrays. C. Cases used for Ki-67 and TUNEL staining.
(XLSX)

Table S2 **Primer sequences used for qPCR.**
(XLSX)

Table S3 **87 genes selected for survival analysis.**
(XLSX)

Table S4 A. Kegg Gene Sets up-regulated in Mets. B. Kegg Gene Sets down-regulated in Mets. C. Reactome Gene Sets up-regulated in Mets. D. Reactome Gene Sets down-regulated in Mets. E. Transcription factor target gene sets up-regulated in Mets. F. Transcription factor target gene sets down-regulated in Mets.
(XLSX)

Table S5 **Percent cancer cells with positive Ki-67 and TUNEL staining.**
(XLSX)

Table S6 **Functional enrichment of top 250 genes correlated with changes in Ki-67 staining between primary and metastatic tumors.**
(XLSX)
